# Understanding the functions of endogenous DOF transcript factor in *Chlamydomonas reinhardtii*

**DOI:** 10.1186/s13068-019-1403-1

**Published:** 2019-03-27

**Authors:** Bin Jia, Xinfeng Xie, Min Wu, Zijie Lin, Jianbo Yin, Sulin lou, Ying Huang, Zhangli Hu

**Affiliations:** Guangdong Engineering Research Center for Marine Algal Biotechnology, Guangdong Key Laboratory of Plant Epigenetics, Shenzhen Key Laboratory of Marine Bioresource and Eco-environmental Science, Longhua Innovation Institute for Biotechnology, College of Life Sciences, Shenzhen University, Shenzhen, 518060 People’s Republic of China

**Keywords:** DOF transcription factor, Lipids, Biofuel, *Chlamydomonas reinhardtii*

## Abstract

**Background:**

The regulation of genes related to lipid metabolism by genetic engineering is an important way to increase the accumulation of lipids in microalgae. DNA binding with one finger (DOF) is a plant-specific transcription factor in higher plants, where it regulates carbon and nitrogen metabolic pathways by regulating key genes involved in these pathways. Overexpression of DOF can increase lipid production in plants; however, it is not clear whether overexpression of DOF can increase lipids in microalgae.

**Results:**

In this study, we cloned a DOF transcription factor, crDOF, from *Chlamydomonas reinhardtii*. The sequence of this transcription factor is 1875 bp and encodes a peptide of 624 amino acids with a conserved DOF domain. Overexpression of crDOF in *C. reinhardtii* significantly increased the intracellular lipid content. The content of total fatty acids in the transgenic algae line Tran^c-crDOF^-12 was 126.01 μg/mg (dry weight), which was 23.24% higher than that of the wild type. Additionally, the content of unsaturated fatty acids in the transgenic Tran^c-crDOF^-12 line increased significantly. Fluorescence quantitative PCR analysis showed that in the transgenic line Tran^c-crDOF^-12, the expression levels of BCC1, FAT1, SQD1, MGD1, DGD1 and PGP1 genes were significantly upregulated, while the expression levels of ACP1, ACS1, CIS1 and SQD2 were downregulated.

**Conclusions:**

Our results confirm that crDOF increases intracellular lipids in *C. reinhardtii* by regulating key genes involved in lipid metabolism. According to these findings, we propose that enhancing the lipid content in microalgae by overexpressing DOF may be achieved in other industrial strains of microalgae and be employed for the industrial production of biodiesel.

**Electronic supplementary material:**

The online version of this article (10.1186/s13068-019-1403-1) contains supplementary material, which is available to authorized users.

## Introduction

Third-generation biodiesel technology with microalgae lipids as the raw material has received widespread attention. Compared with other oil crops, microalgae have the advantage of fast growth, low culture cost, and high photosynthetic and lipid production efficiency. More importantly, microalgae are not limited by cultivating land, irrigation and nutrition and can effectively convert solar energy into lipids [1, 2]. However, the natural microalgae lipid content is limited, resulting in a higher price for microalgae biodiesel. Therefore, increasing the amount of lipids in microalgae is the key issue in the current study.

In recent years, genetic engineering has been widely used to improve the amount of lipids in microalgae [3, 4]. Related studies have focused on the overexpression of key enzymes in fatty acid synthesis [5] or in triacylglyceride synthesis [6] and on knockout or inhibition of enzymes that compete with lipid accumulation [7, 8]. Most of the studies concentrated on single-gene/enzyme regulation. However, the synthesis and consumption of lipids are a complex metabolic network, rather than a single path level, in vivo. Multiple gene regulation should be preferred. Thus, the idea of multigene regulation by transcription factors provides a new choice and may greatly reduce the cost in the future.

DOF (DNA binding with one finger) transcription factors are plant-specific transcription factors. These transcription factors contain a single Cys-rich zinc finger DNA-binding domain that can specifically recognise the plant promoter and thereby activate or inhibit plant gene expression. In higher plants, DOF transcription factors are involved in a variety of biological processes, including photoreaction and photosynthetic pigment response, seed development and germination, carbon and nitrogen metabolism, and defence functions. More importantly, studies have shown that plant DOF transcription factors are also involved in lipid metabolism. Overexpression of a soybean DOF transcription factor in *Arabidopsis* increased the total lipid content in seeds [9]. Overexpression of a soybean DOF transcription factor in *Chlorella ellipsoidea* increased cell lipid content by 46.4–52.9% [10]. However, little is known about DOF transcription factors in microalgae. Only a potential ancestral DOF gene, *crDOF*, was discovered in *C. reinhardtii* [11]. However, the specific function of this gene is unclear, and it is also unknown whether it regulates lipid metabolism. To explore the relationship between crDOF and lipid metabolism, in this study, we cloned crDOF, overexpressed it in *C. reinhardtii*, and analysed the intracellular lipid content in the transgenic cell lines. Furthermore, we suggested that crDOF enhances lipid accumulation by modulating key genes involved in lipid metabolism through fluorescent quantitative RT-PCR.

## Results

### Analysis of the DOF gene in *C. reinhardtii*

**Fig. 1:**
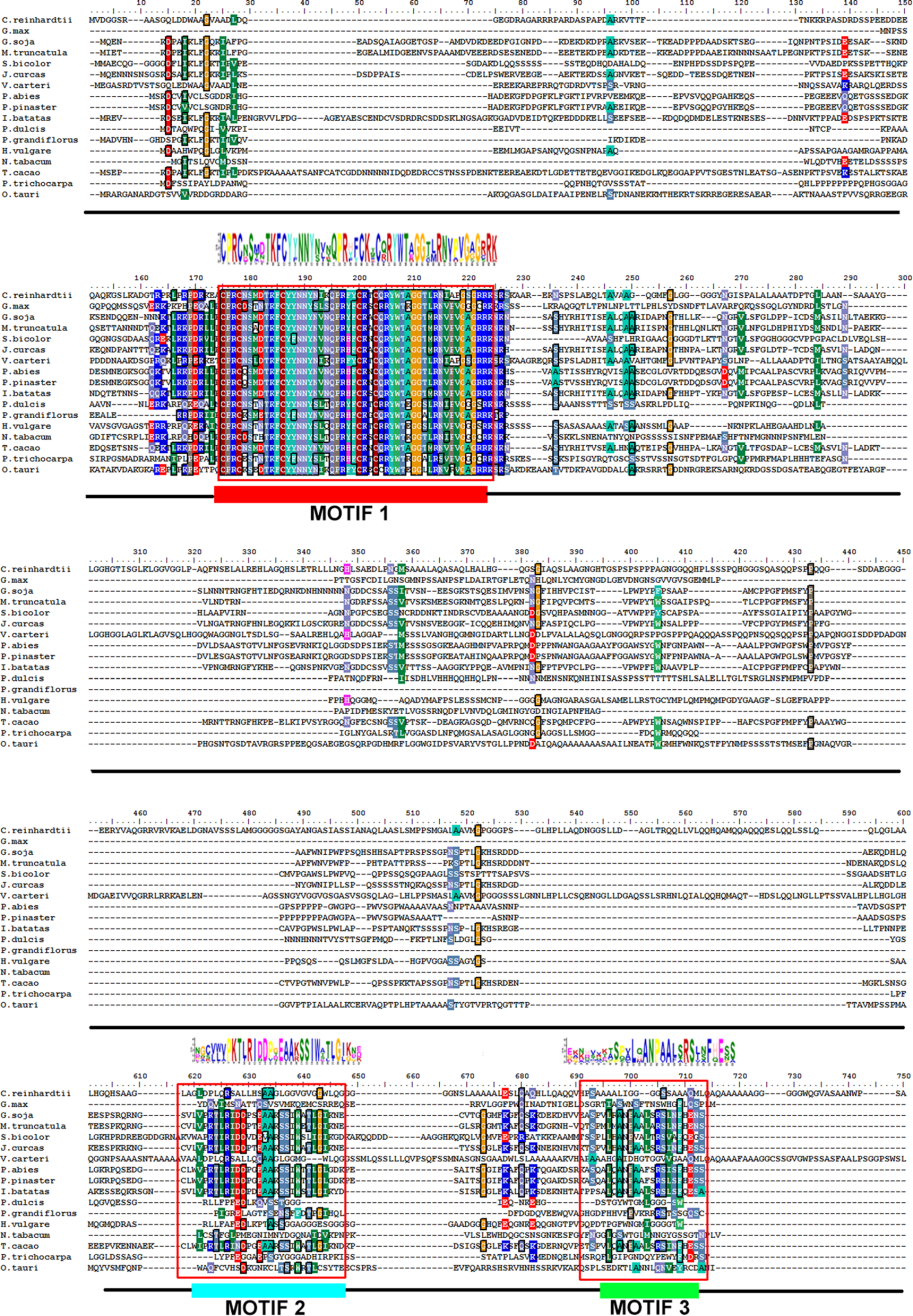
Multiple alignment of DOF proteins from *C. reinhardtii*, *G. max*, *V. carteri*, *G. soja*, *M. truncatula*, *S. bicolor*, *J. curcas*, *P. abies*, *P. pinaster*, *I. batatas*, *P. dulcis*, *P. grandiflorus*, *H. vulgare*, *N. tabacum*, *T. cacao* and *P. trichocarpa*. The red box within the alignment and corresponding sequence logo denotes the conserved motifs identified by the MEME programme

**Fig. 2:**
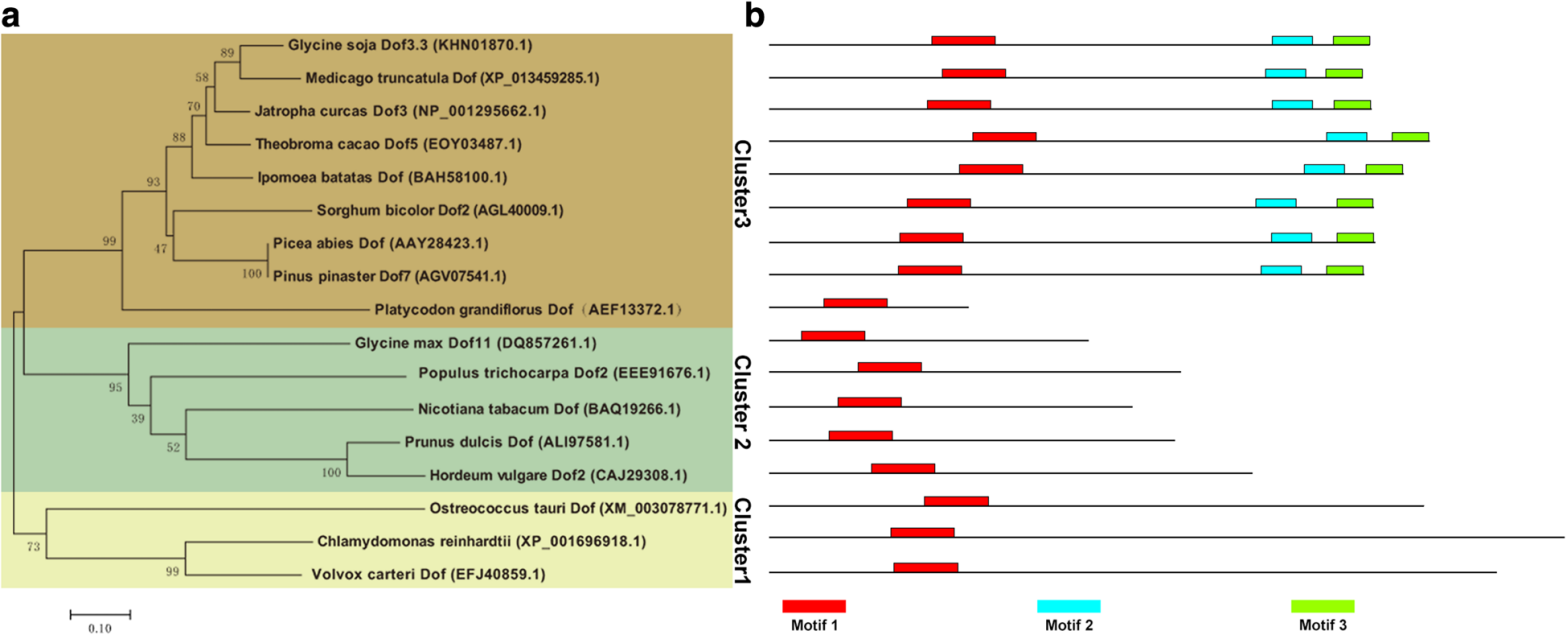
**a** Phylogenetic analysis of crDOF with DOFs from *G. max*, *V. carteri*, *G. soja*, *M. truncatula*, *S. bicolor*, *J. curcas*, *P. abies*, *P. pinaster*, *I. batatas*, *P. dulcis*, *P. grandiflorus*, *H. vulgare*, *N. tabacum*, *T. cacao* and *P. trichocarpa* (sequence IDs listed in Additional file 5: Table S2). The tree was constructed using the neighbour-joining algorithm. Numbers at branch points are bootstrap percentages derived from 1000 replicates. **b** The MEME motifs are shown in different coloured boxes

There is only one annotated DOF transcription factor gene in C. *reinhardtii*, which is located on chromosome 12 and has a length of 3.83 kb. The cDNA, *crDOF*, was successfully cloned by RT-PCR with the primers listed in Table 1. The *crDOF* is 1875 bp and encodes 624 amino acids, which is identical to the sequence of Cre12.g521150 in the genome. Multiple alignment of the deduced crDOF encoding protein with other known plant DOF proteins showed that a highly conserved region of 50 amino acids was located in the N-terminal region (Fig. 1). In this region, four conserved Cys residues form a C(X)_2_C(X)_21_C(X)_2_C motif that can form a single zinc finger-like structure together with Zn^2+^. In addition, this sequence contains Tyr and Trp residues necessary for DNA binding. Furthermore, MEME was used to analyse the distribution of domains in all the DOF sequences, as shown in Figs. 1 and 2. Three motifs were found after MEME analysis. Motif 1, representing the DOF domain, was present in all selected proteins and located in the N-terminal conserved region using multiple alignment. Motif 2, representing the GI-binding domain, and motif 3, representing the FKF1-binding domain, were found at the C-terminus. However, as shown in Fig. 2, motif 2 and motif 3 were not found in the DOF sequences from *C. reinhardtii*, *G. max*, *V. carteri*, *P. dulcis*, *H. vulgare*, *O. taur* and *P. trichocarpa.*

**Table 1:** The content of fatty acid components in wild-type and transgenic lines

Fatty acid	Wild type (μg/mg DW)	Tran^c-crDOF^-12 (μg/mg DW)	Increase (%)	Tran^c-gDOF^-60 (μg/mg DW)	Increase (%)
C16:0	32.38 ± 0.12	41.37 ± 0.13*	27.79	42.11 ± 0.01*	30.07
C16:1	0.79 ± 0.01	4.09 ± 0.11**	416.46	4.58 ± 0.04**	478.26
C18:0	7.52 ± 0.06.	5.72 ± 0.01	− 23.94	8.25 ± 0.07	9.75
C18:1 t	9.95 ± 0.11	5.30 ± 0.19^#^	− 46.781	15.37 ± 0.19*	54.44
C18:1 c	4.98 ± 0.13	9.27 ± 0.15*	86.07	15.26 ± 0.18**	206.36
C18:2 t	13.84 ± 0.02	13.96 ± 0.04	0.89	14.40 ± 0.22	4.05
C18:3n6	32.80 ± 0.22	47.40 ± 0.03**	44.53	37.54 ± 0.27*	14.48

* Significant increase (*p* < 0.05) compared with control strain cc849. ** Extremely significant increase (*p* < 0.01) compared with control strain cc849

^#^Very significant decline (*p* < 0.05) compared with control strain cc849

The DOF protein phylogenetic tree was constructed with Mega 6.0 using the neighbour-joining method. As shown in Fig. 2, DOF proteins were divided into three clusters. Cluster 1 was made up of algal DOF proteins derived from *C. reinhardtii*, *V. carteri* and *O. taur*, and they only contained motif 1. Clusters 2 and 3 consisted of DOF proteins derived from higher plants. DOF proteins in cluster 2 contained motif 1 only, while DOF proteins in cluster 3 contained all three motifs. In this phylogenetic tree, crDOF was most closely related to *V. carteri* DOF. The sequence identity between them was 82%. From the above results, we inferred that crDOF is the endogenous DOF transcription factor in *C. reinhardtii*.

### Overexpression of the DOF transcription factor in *C. reinhardtii*

Previous reports have shown that overexpression of the soybean DOF transcription factor in *C. reinhardtii* can increase the intracellular lipid content [11]. As a result, whether overexpression of crDOF can increase lipid accumulation was studied. To this end, the soybean DOF was selected as a positive control. Codon analysis showed that the average difference in codon usage between soybean and *C. reinhardtii* was 54.58% and that the difference is more obvious in codons containing C and G only (GCG, CGC, etc.). Therefore, we optimised the soybean DOF gene according to the codon preference of *C. reinhardtii*. The optimised gene, gDOF (accession number: XP_001696918.1), has a G + C content of 70.8% and a CAI value of 0.616, which is more consistent with the codon preference of nuclear *C. reinhardtii*. The codon usage of gDOF compared with non-optimised soybean DOF is shown in Additional file 1: Figure S1.

**Fig. 3:**
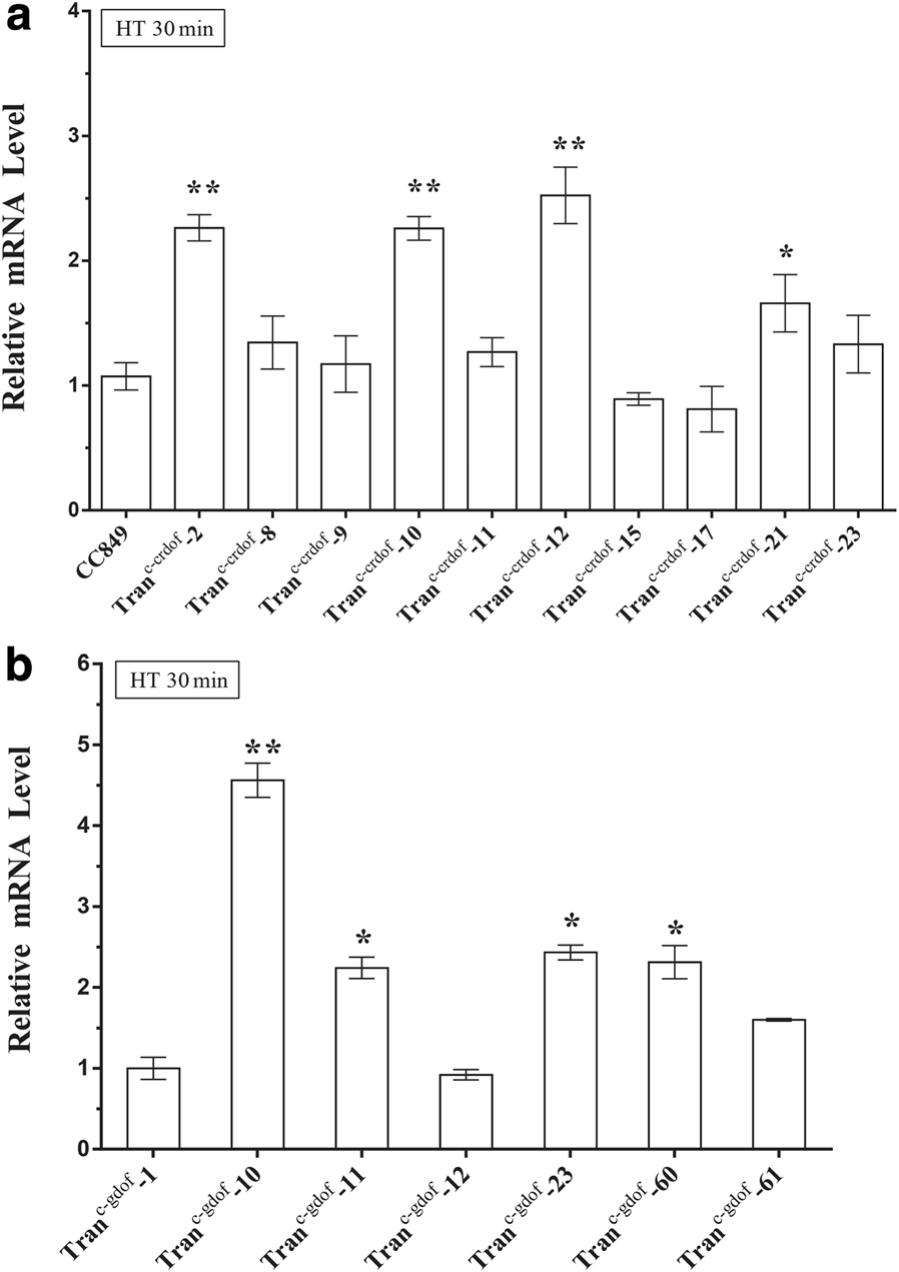
The mRNA levels of the DOF transcription factor gene in transgenic microalgae. (*) indicates a significant difference (P < 0.05) compared to the control strain; (**) indicates an extremely significant difference (P < 0.01) compared to the control strain. (**a** The mRNA levels of crDOF were clearly different among transformants. **b** The mRNA levels of gDOF were significantly different among different transformants of *C. reinhardtii.*)

To overexpress crDOF and gDOF in *C. reinhardtii*, both genes were inserted separately downstream of the heat-inducible HSP70-RBCS2 promoter in the vector pJDHSP and then transformed into *C. reinhardtii*. After 3 weeks, about 50 transformants of each type were randomly selected for verification the integration of DOF expression unit. Because random integration of an exogenous gene in *C. reinhardtii* often results in varied expression levels, about 20 PCR-verified transformants were selected to screen for relatively higher mRNA levels by qRT-PCR. Compared with wild type, most transformants showed higher mRNA expression levels with or without heat induction. Moreover, heat induction significantly increased the expression of mRNA. As shown in Fig. 3, the mRNA levels of crDOF and gDOF were significantly different among transformants after heat induction. The mRNA levels of crDOF in Tran^c-crDOF^ transformants # 2, 10, 12, and 21 all showed a more than twofold increase over that of wild-type cc849 after heat induction. Among them, Tran^c-crDOF^-12 exhibited the highest mRNA level, which was almost a 2.5-fold increase over that of cc849. Similarly, the Tran^c-gDOF^ transformants # 10, 11, 23 and 60 showed high mRNA levels. Among them, Tran^c-gDOF^-10 showed the highest mRNA level, which was 4.1-fold higher than that of Tran^c-gDOF^-12 which showed the lowest mRNA level.

### Analysis of lipid accumulation in transgenic microalgae

**Fig. 4:**
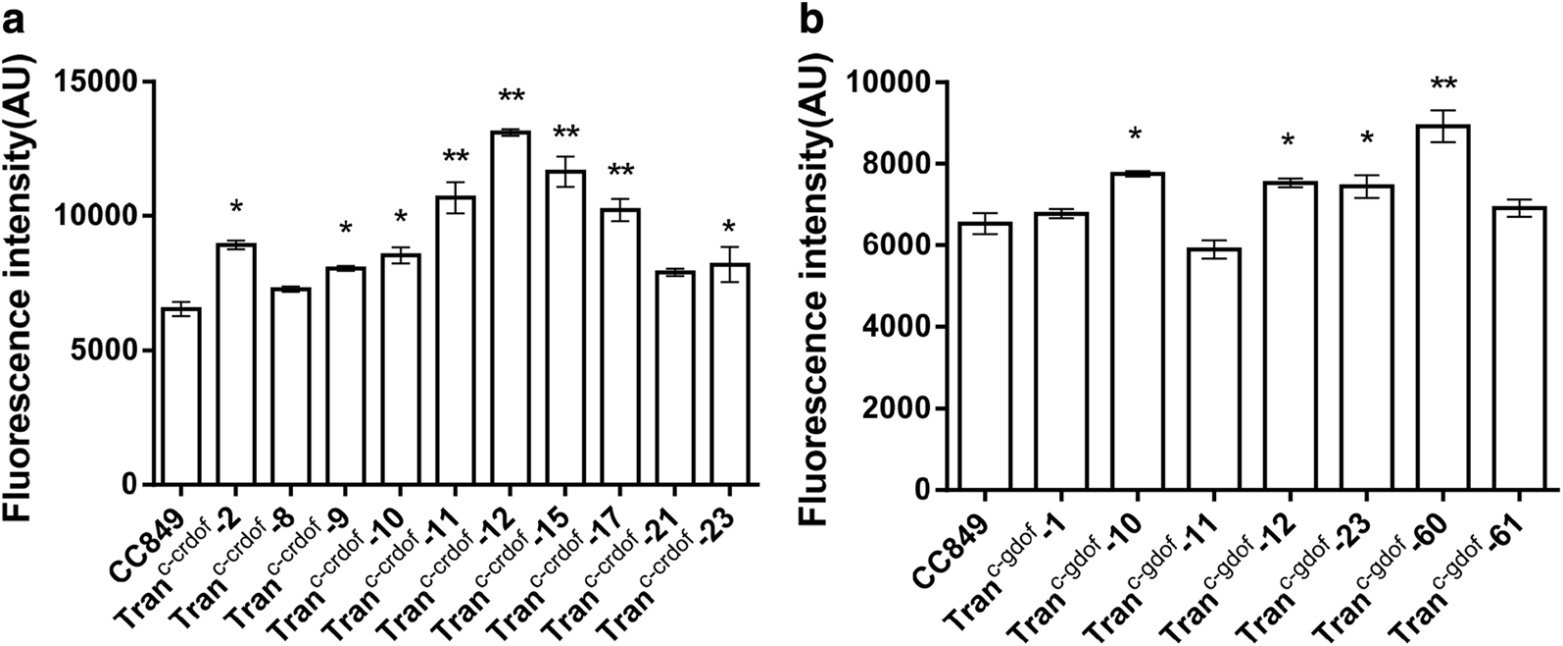
Rapid analysis of lipids content in transgenic microalgae. (*) indicates a significant difference (*p* < 0.05) compared to the control strain; (**) indicates an extremely significant difference (*p* < 0.01) compared to control strain cc849

BODIPY 505/515 can specifically stain intracellular neutral lipids and shows green fluorescence under blue light excitation. The fluorescence intensity of BODIPY is positively correlated with the intracellular lipid content [12]. Therefore, the BODIPY staining method was used to quantify the neutral lipids in transgenic algae. As shown in Fig. 4, most transgenic algae exhibited higher fluorescence intensity after BODIPY staining than wild-type cc849. The fluorescence intensity of Tran^c-crDOF^ transformants # 11, 12, 15, and 17 showed a significant increase (*p* < 0.01), among which Tran^c-crDOF^-12 showed the highest fluorescence intensity, being 1.8-fold higher than that of the wild-type strain. The fluorescence intensity of Tran^c-gDOF^ transformants # 10, 12, 23 and 60 showed a significant increase (*p* < 0.01). The highest fluorescence intensity was found in Tran^c-gDOF^-60, which showed a 1.5-fold higher fluorescence intensity than that of the wild-type strain. And after induction, the transformants even showed higher fluorescence intensity over time (Additional file 2: Figure S2). These data indicated that crDOF actually affected the neutral lipids, mainly TAG, content, and overexpression of crDOF can obviously increase intracellular TAG in *Chlamydomonas reinhardtii*. To further explore the specific effects of DOF transcription factors, transgenic Tran^c-crDOF^-12 and Tran^c-gDOF^-60 were selected for subsequent experiments.

The growth curves of transgenic Tran^c-crDOF^-12 and Tran^c-gDOF^-60 are shown in Additional file 3: Figure S3. Compared with wild-type cc849, their growth rate did not show a significant difference, indicating that crDOF and gDOF did not influence the growth of *C. reinhardtii*. The fatty acid content and components of these two transgenic algae were analysed by GC–MASS. Three replicates were used for each strain. The results showed that the main fatty acids in the transgenic algae were C16:0 (palmitic acid), C16:1 (palmitoleic acid), C18:0 (stearic acid), C18:1t (oleic acid), C18:1c (methyl oleate), C18:2t (linoleic acid) and C18:3n6 (methyl linolenate).

**Fig. 5:**
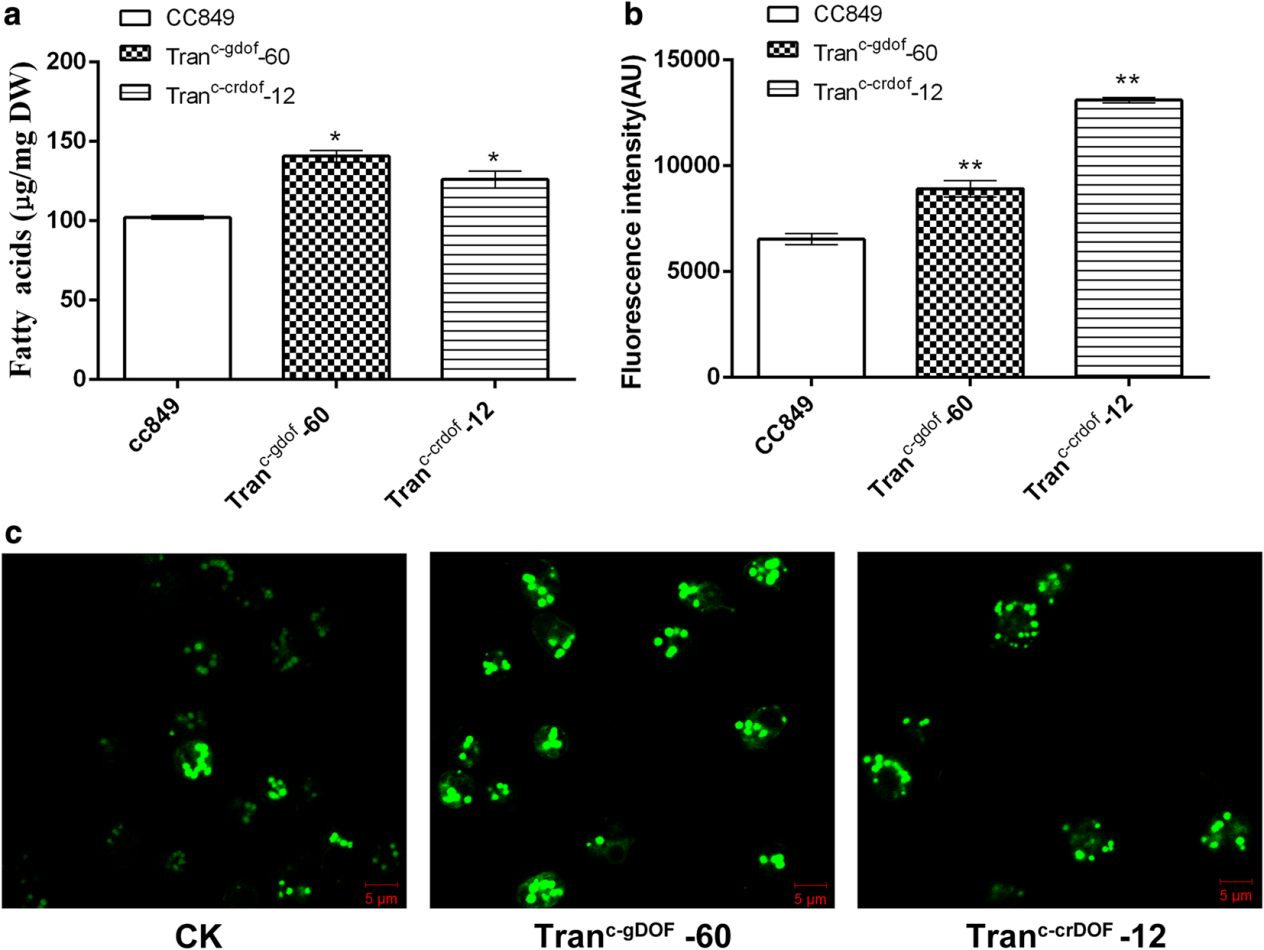
The content of total fatty acids (**a**) and neutral lipids (**b**) in control strain cc849 and transgenic lines Tran^c-crDOF^-12 and Tran^c-gDOF^-60. (*) indicates a significant difference (*p* < 0.05) compared to the control strain; (**) indicates an extremely significant difference (*p* < 0.01) compared to control strain cc849. **c** Transgenic lines Tran^c-crDOF^-12 and Tran^c-gDOF^-60 accumulated more intracellular lipids. Intracellular lipids droplets stained with BODIPY 505/515 were observed by a confocal microscope. The scale bar represents 5 μm

As shown in Fig. 5a, the total fatty acids of heat-induced Tran^c-crDOF^-12, Tran^c-gDOF^-60 and wild-type cc849 were 126.01 μg/mg, 137.94 μg/mg and 102.32 μg/mg DCW (dry cell weight), respectively, which were 23.2 and 30.1% higher than that of cc489, with a significant difference (P < 0.01). The neutral lipid content of Tran^c-crDOF^-12 and Tran^c-gDOF^-60 was approximately 2- and 1.36-fold higher that of cc849, as characterised by BODIPY staining (Fig. 5b). Intracellular fatty acid profiles of Tran^c-gDOF^-60, Tran^c-crDOF^-12 and cc849 were analysed by GC–MASS. As shown in Table 1, although the most prominent fatty acids in all microalgae remained C16:0, C18:1 and C18:3, their content had changed considerably when compared to that of cc849. The content of fatty acids C16:1 and C18:3n6 was increased significantly (*p* < 0.01) in Tran^c-crDOF^-12, with increases of 416.46% and 44.53%, respectively. Similarly, the content of fatty acids C16:1 and C18:1c increased significantly (*p* < 0.01) in Tran^c-gDOF^-60, with increases of 478.26% and 206.36%, respectively. In other words, the content of intracellular unsaturated fatty acids increased significantly in transgenic algae Tran^c-gDOF^-60 and Tran^c-crDOF^-12.

The intracellular lipid droplet distribution in algae was stained by BODIPY505/515, was visualised by a laser confocal microscope and is shown in Fig. 5c. The brightness and number of lipid droplets in transgenic Tran^c-crDOF^-12 and Tran^c-gDOF^-60 were stronger and higher than those in cc849. In addition, lipid droplets of transgenic strains were larger than those of cc849. These results showed that crDOF had a function in lipid synthesis similar to that of soybean DOF transcription factor and that overexpression of crDOF in *C. reinhardtii* could significantly increase the amount of intracellular lipids and the content of unsaturated fatty acids.

### Quantitative analysis of key genes in lipid synthesis

**Fig. 6:**
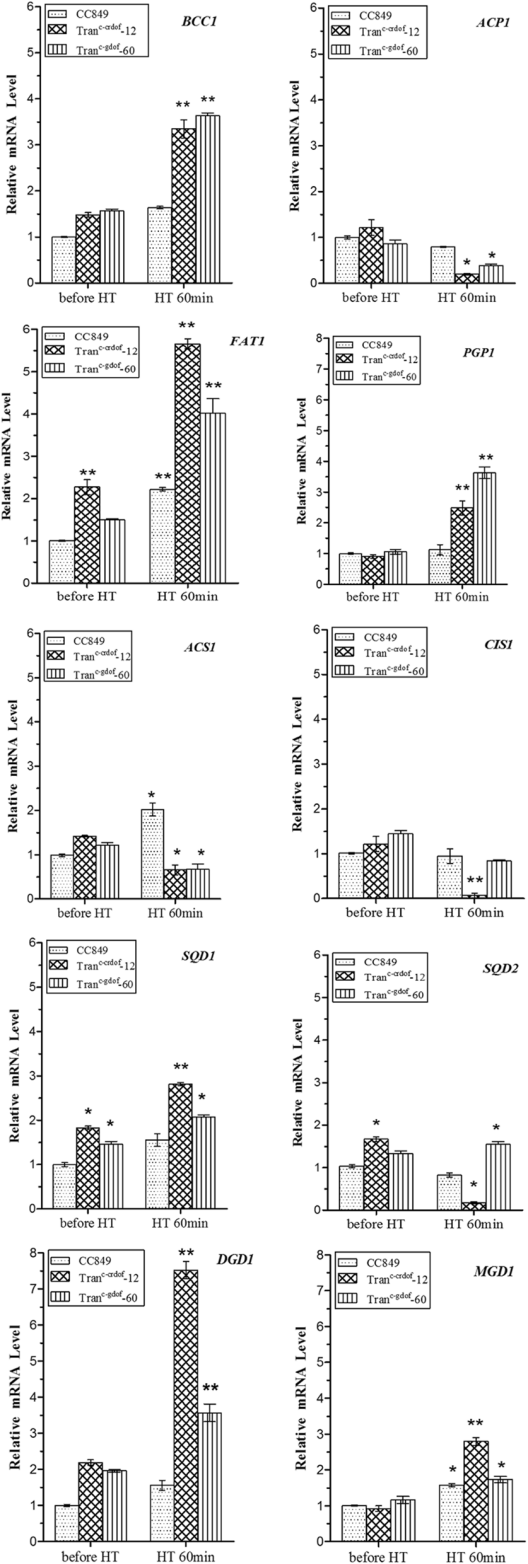
Expression profiles of ten genes that have effects on lipid metabolism in transgenic algae Tran^c-crDOF^-12 and Tran^c-gDOF^-60 and control strain cc849 by quantitative real-time (qRT-PCR). * indicates a significant difference (*p* < 0.05) compared to the control strain; ** indicates an extremely significant difference (*p* < 0.01) compared to the control strain. *BCC1* acetyl-coA biotin carboxyl carrier 1, *ACP1* acyl-carrier protein, *FAT1* fatty acyl–acyl carrier protein thioesterase, *ACS1* acetyl CoA synthetase, *CIS1* citrate synthase, *SQD1* UDP-sulfoquinovose synthase, *SQD2* sulfolipid synthase, *MGD1* monogalactosyldiacylglycerol synthase, *DGD1* digalactosyldiacylglycerol synthase, *PGP1* phosphatidyl glycerophosphate synthase

The above results showed that overexpression of crDOF could increase intracellular lipids in *C. reinhardtii*. To further explore the reasons for this increase, the mRNA levels of 10 key enzyme genes (*BCC1, ACP1, SQD1, SQD2, MGD1, DGD1, FAT1, CIS1, ACS1* and *PGP1*), which play key roles in the lipid metabolism of *C. reinhardtii*, were analysed. As shown in Fig. 6, the mRNA levels of key genes involved in lipid metabolism in transgenic lines exhibited distinct profiles when compared to those in wild-type cc849. The changes in mRNA levels are summarised in Table 2. The mRNA levels of *BCC1, FAT1, SQD1, MGD1, DGD1* and *PGP1* in Tran^c-crDOF^-12 were significantly upregulated to 2.04, 2.55, 1.81, 1.77, 4.48 and 2.21 times those in the wild type, respectively. The mRNA levels of *ACP1, ACS1, CIS1* and *SQD2* genes were downregulated to 0.25-, 0.33-, 0.07- and 0.21-fold those in the wild type, respectively. Similar changes were found in Tran^c-gDOF^-60, where the mRNA levels of *BCC1, FAT1, SQD2, DGD1* and *PGP1* were 2.22, 1.81, 1.88, 2.29 and 3.23 times those in the wild type, while the mRNA levels of *ACP1* and *ACS1* decreased to 0.49 and 0.33 times those in the wild type. However, the mRNA levels of *CIS*, *SQD1* and *MGD1* did not change significantly. Overall, the mRNA levels of *BCC1, FAT1, DGD1* and *PGP1* in transgenic lines increased significantly, while the mRNA levels of *ACP1* and *ACS1* genes were downregulated. Among the upregulated genes, *BCC1* is a component of the acetyl coenzyme A carboxylase complex, which catalyses the first step of fatty acid synthesis. FAT1 is a distinctive domain within a large multifunctional fatty acid synthase polyprotein, which plays a key role in fatty acid elongation, while *DGD1* and *PGP1* are involved in glyceride synthesis. These data suggest that crDOF increased the mRNA levels of key enzyme genes in fatty acid and glyceride synthesis pathways in *C. reinhardtii*. We also found that the mRNA level of long-chain acyl-CoA synthase *ACS1*, which is responsible for free fatty acid activation and degradation, was downregulated. This finding indicates that crDOF may affect the degradation of fatty acids in vivo. In summary, we believe that crDOF enhanced intracellular lipid accumulation by influencing lipid metabolism-related genes.

**Table 2:** Fold changes in mRNA levels of lipid metabolism-related genes in transgenic algae

Gene	Tran^c-crDOF^-12	Tran^c-gDOF^-60
*BCC1*	↑ 2.04*	↑2.22**
*ACP1*	↓ 0.25^##^	↓ 0.49^#^
*FAT1*	↑2.55**	↑1.81*
*ACS1*	↓0.33^#^	↓ 0.33^#^
*CIS1*	↓ 0.07^#^	NC
*SQD1*	↑1.81*	NC
*SQD2*	↓0.21^##^	↑1.88*
*MGD1*	↑1.77**	NC
*DGD1*	↑ 4.84**	↑ 2.29**
*PGP1*	↑ 2.21**	↑ 3.23**

* Significant increase compared with control strain cc849 (*p* < 0.05). ** Extremely significant increase (*p* < 0.01) compared with control strain cc849

^#^Significant decrease (*p* < 0.05) compared with control strain cc849. ^##^Extremely significant decrease (*p* < 0.01) compared with control strain cc849. NC: no significant change

### Hyperaccumulation of lipids in transgenic algae by multiple rounds of heat induction

**Fig. 7:**
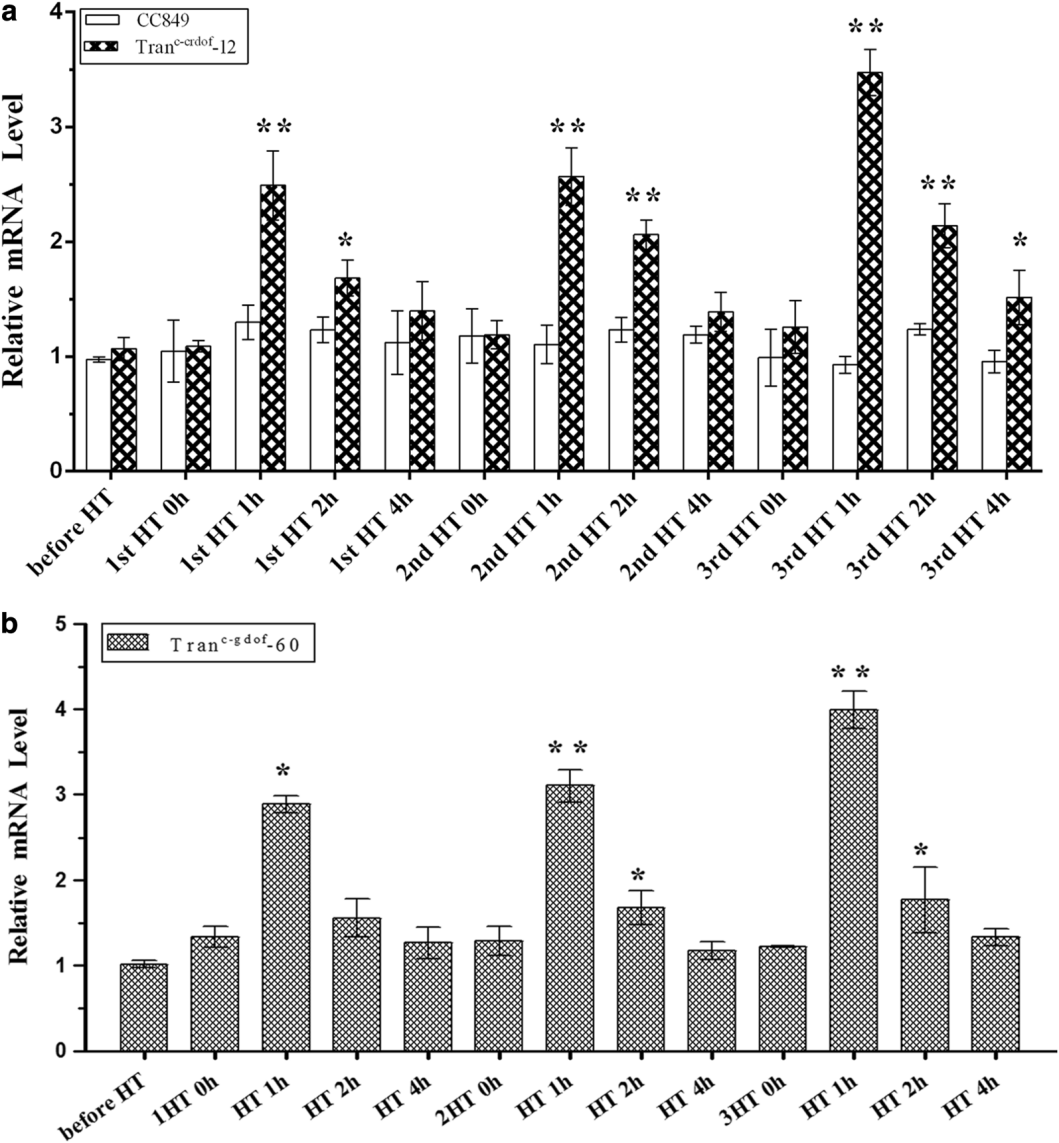
Multiple heat inductions increase the mRNA levels of the genes crDOF and gDOF. (*) indicates a significant difference (*p* < 0.05) compared to the control strain; (**) indicates an extremely significant difference (*p* < 0.01) compared to control strain cc849

The heat-inducible promoter HSP70-RBCS2 can be induced multiple times for enhanced protein overexpression. Thus, the effect of three rounds of heat induction on lipid accumulation in transgenic lines was further investigated. The mRNA levels of crDOF in Tran^c-crDOF^-12 at 0 h, 1 h, 2 h and 4 h after each round of heat induction are shown in Fig. 7a. In each round of heat induction, a peak could be found at 1 h, and the mRNA levels gradually decreased with the elongation of recovery time. Thus, a wave-like mRNA expression pattern appeared during the whole process of three rounds of heat induction. The three peak mRNA levels of crDOF in Tran^c-crDOF^-12 after the first, second and third rounds of heat induction were 2.52-, 2.56- and 3.51-fold higher than those in the control, respectively. Similar results were also found in the transgenic line Tran^c-gDOF^-60 (Fig. 7b) with 2.88-, 3.21-, and 4.12-fold increases in the mRNA levels of gDOF after each round of heat induction.

After multiple rounds of heat induction, the transgenic algae obviously accumulated more lipids. The total lipid content in Tran^c-crDOF^-12, Tran^c-gDOF^-60 and cc849 was 179.82 μg/mg, 201.19 μg/mg and 118.42 μg/mg DCW, respectively, which showed increases of 51.85% and 69.89% over that of the control strain. The intracellular fatty acid profiles of Tran^c-gDOF^-60, Tran^c-crDOF^-12 and cc849 are listed in Table 3. The content of common kinds of fatty acids, such as C16:0 and C18:1, in both transgenic algae showed a significant increase. This finding indicated that the expression of crDOF in a transgenic line could be regulated by multiple rounds of heat induction, which could further increase the intracellular lipid content.

**Table 3:** The content of fatty acid components in wild-type and transgenic lines after multiple heat inductions

Fatty acid	Wild type (µg/mg DW)	Tran^c-crdof^-12 (µg/mg DW)	Increase (%)	Tran^c-gdof^-60 (µg/mg DW)	Increase (%)
C16:0	38.71 ± 0.08	58.84 ± 0.02**	52.01	69.49 ± 0.06**	79.52
C16:1	2.53 ± 0.03	3.84 ± 0.13	52.13	3.44 ± 0.12	36.09
C18:0	5.44 ± 0.16.	8.26 ± 0.09	51.97	4.46 ± 0.08	− 18.09
C18:1 t	12.19 ± 0.09	18.53 ± 0.29	51.98	28.86 ± 0.01**	135.13
C18:1 c	12.10 ± 0.07	19.39 ± 0.18	53.21	28.45 ± 0.21**	135.11
C18:2 t	13.53 ± 0.23	19.57 ± 0.03*	50.01	14.32 ± 0.09	5.82
C18:3n6	33.80 ± 0.19	51.38 ± 0.25*	52.01	52.37 ± 0.17*	54.95

* Significant increase (P < 0.05) compared with control strain cc849. ** Extremely significant increase (*p* < 0.01) compared with control strain cc849

^#^Very significant decline (P < 0.05) compared with control strain cc849

## Discussion

Increasing the content of intracellular lipids by genetic engineering has always been the core problem of microalgae energy research. To date, strategies for enhancing lipid accumulation by genetic engineering in microalgae can be mainly classified into three categories. The first category is the regulation of key genes that are directly involved in lipid metabolism pathways, such as de novo synthesis of fatty acids, synthesis of triglycerides, and beta-oxidation of fatty acids. The most common regulated genes include ACCase (the first rate-limiting enzyme in de novo fatty acid synthesis), fatty acid synthase (FAS, responsible for fatty acid chain extension), 3-phosphoglycerol dehydrogenase (G3PDH), glycerol-3-phosphate acyltransferase (GPAT), lysophosphatidic diacyltransferase (LPDAT), phospholipid:diacylglycerol acyltransferase (PDAT), phosphatidate phosphatase (PAP), and diacylglycerol acyltransferase (DGAT) [13–18]. The second category is the regulation of genes not directly involved in, but relevant to, lipid metabolism. In this method, the regulated genes are often related to starch synthesis, glucose metabolism, the citric acid cycle and amino acid metabolism, and examples of these genes include ADP-glucose pyrophosphorylase (AGPase) [19], phosphoenolpyruvate carboxylase [8] (PEPC), citrate synthase (CIS) [20] and malic enzyme (ME) [6]. The third category is the regulation of global factors, for example, transcription factors. Thus far, the reported transcription factors involved in lipid metabolism are DOF, SNF2, bHLH and PHR1 [9, 21–23]. In general, the first two categories mainly involve single-gene regulation, which may have a poor or even no effect. For example, overexpression of the ACCase gene in a diatom (*C. cryptica*), despite increased ACCase activity, did not result in a significant increase in lipid content [24]. Notably, the method of global regulation by transcription factors that can alter the expression of multiple enzymes has been emphasised recently.

Lipid enhancement by DOF transcription factors was first discovered in plants and later in microalgae. Overexpression of the soybean DOF transcription factor GmDOF4 or GmDOF11 in *Arabidopsis* can increase the lipid content in seeds [9]. Subsequent studies showed that overexpression of GmDOF4 in *C. ellipsoidea* could increase the intracellular lipid content by 46.4–52.9% without affecting its growth rate and could increase the expression levels of acetyl-CoA carboxylase [10]. In addition, overexpression of GmDOF11 in *C. reinhardtii* increased its lipid content by 1.8–2.3-fold [11]. These studies confirmed that the soybean DOF can increase lipid content, but whether microalgae DOF transcription factors, especially those of *C. reinhardtii*, can enhance lipids is still unclear. In this paper, we cloned the DOF transcription factor gene from *C. reinhardtii*, over-expressed it in *C. reinhardtii* and confirmed that crDOF could increase intracellular lipid content. The contents of total fatty acids and neutral lipids in the transgenic lines were increased by 23.24% and 200% when compared to those of the control cc849. To the best of our knowledge, this is the first study to increase intracellular lipids in microalgae by regulating its native DOF transcription factor.

DOF transcription factors can recognise the AAAG or CTTT motif in the promoter region of their binding genes [25]. We find that these motifs are present in the promoter regions of ten selected genes (*BCC1*, *ACP1*, *SQD1*, *SQD2*, *MGD1, DGD1*, *FAT1*, *CIS1*, *ACS1*, and *PGP1*) associated with lipid metabolism. *BCC1* is the biotinylated catalytic subunit of ACCase, which catalyses the first step of fatty acid synthesis, acetyl-CoA to malonyl-CoA. This step is the first rate-limiting step of fatty acid synthesis. Overexpression of BCC1 resulted in greater immobilisation of CO_2_ and increased carbon flow to fatty acid synthesis [25]. In this study, expression of crDOF or gDOF led to a 2.1- or 2.2-fold increase in the mRNA levels of BCC. Similar results were found in *C. ellipsoidea*, where expression of GmDOF4 increased the mRNA level of BCC by 6.95- to 8.41-fold [10]. We also found that the mRNA levels of the fatty acid synthesis gene *FAT1* and of the triglyceride synthesis genes *DGD1* and *PGP1* were significantly upregulated by crDOF, with increases of 2.55-, 4.84- and 2.21-fold, respectively. On the other hand, the mRNA levels of *CIS1* and *ACS1* were downregulated by crDOF. *CIS1* is the first and rate-limiting step in the tricarboxylic acid cycle, catalysing the generation of citrate coenzyme A [20] found that knockdown of *CIS1* in *C. reinhardtii* could increase its TAG content by 169.5%, while overexpression of *CIS1* could reduce the TAG content by 45%. In our study, the mRNA level of *CIS1* in Tran^c-crDOF^-12 was reduced by 0.92-fold compared with that in the wild type, suggesting that crDOF might also increase lipids by repression of *CIS1*. *ACS* can activate fatty acids into CoA thioesters, which can then serve as the substrates for many metabolic pathways. Interestingly, our previous research confirmed that downregulation of *ACS1* in *C. reinhardtii* could increase the lipid content by 45% [26]. Here, we found that the mRNA level of *ACS1* in Tran^c-crDOF^-12 was approximately threefold lower than that in the wild type, indicating that the increase in intracellular lipids could also result from crDOF-induced *ACS1* repression.

## Conclusions

We cloned the transcription factor crDOF and constructed transgenic lines of *C. reinhardtii* with increased intracellular lipids by overexpressing crDOF. Furthermore, we confirmed that crDOF increased the intracellular lipids of *C. reinhardtii* by regulating key genes involved in lipid metabolism. According to our findings, we propose that enhancing lipids in microalgae by overexpressing algal DOF may be achieved in other industrial microalgae and be employed for the industrial production of biodiesel.

## Materials and methods

### Strains, vectors and culture conditions

Cell wall-deficient *C. reinhardtii* cc849 (from the Chlamydomonas Resource Centre) was cultured in Tris–acetate–phosphate (TAP) medium under continuous light (60 μmol/m/s) at 25 °C. The vector pJDHSP containing the heat-inducible promoter Hsp70-RBCS2 was used for DOF gene overexpression in *C. reinhardtii*. To induce gene expression, *C. reinhardtii* transformants were first cultured to a density of 1–2×10^6^ cells/mL and then incubated at 40 °C for 30 min. After that, the cells were cultured at 25 °C for 48 h. *E. coli* DH5α cells were used for normal DNA manipulation and cultured in LB medium at 37 °C.

### Cloning and analysis of *crDOF*

The predicted locus of the *crDOF* gene in *C. reinhardtii* was *Cre12.g521150*. According to this sequence, the primers c-crDOF-F and c-crDOF-R are listed in Additional file 4: Table S1 is designed to amplify crDOF. Total RNA of *C. reinhardtii* was isolated with a TaKaRa RNAiso Plus Kit and used for cDNA synthesis with oligo-dT as the reverse transcription primer. RNA of crDOF was amplified using LA Taq polymerase (TaKaRa) with high GC buffer II. The amplification condition was 94 °C for 4 min, followed by 30 cycles of synthesis (94 °C for 30 s, 60 °C for 30 s, and 72 °C for 2 min), and a final extension step at 72 °C for 5 min. PCR products were then cloned into the pEASY-T vector (Transgene) after purification and subjected to sequencing.

The full-length crDOF protein sequence was aligned with other DOFs originating from *Glycine max*, *Volvox carteri*, *Glycine soja*, *Medicago truncatula*, *Sorghum bicolor*, *Jatropha curcas*, *Picea abies*, *Pinus pinaster*, *Ipomoea batatas*, *Prunus dulcis*, *Platycodon grandiflorus*, *Hordeum vulgare*, *Nicotiana tabacum*, *Theobroma cacao* and *Populus trichocarpa* (sequence IDs listed in Additional file 5: Table S2) using ClustalX. Phylogenetic trees were constructed with MEGA 4.0 using a neighbour-joining algorithm with the substitution model JTT + G 0.54. The number of bootstrap replicates was 500. MEME was used to analyse the distribution of domains in all the DOF sequences. The GI-binding and FKF1-binding domains were identified as described previously [27].

### Plasmid construction and transformation

The soybean DOF transcription factor gene *DOF11* (NCBI accession NO: DQ857261.1) was used as a positive control. Due to the large differences in the codon and GC content between *C. reinhardtii* and soybean, we optimised this gene for the nuclear codon bias of *C. reinhardtii* using the online tool Optimizer, and the General Biosystem Company synthesised the gene. The synthesised gene gDOF was then ligated into the vector pJDHSP [28] after *Kpn* I and *Pmac* I digestion to generate the expression plasmid *pJD*-*gDOF*. Similarly, the *C. reinhardtii* native DOF gene, crDOF, was also ligated into pJDHSP after *Kpn* I and *Pmac* I digestion to generate the expression plasmid *pJD*-*crDOF*.

The plasmids pJD-gDOF and pJD-crDOF were separately transformed into CC-849 cells by glass bead aggregation [29]. Briefly, CC-849 cells were grown to a density of 1–2 × 10^6^ cells/mL and then concentrated to 2 × 10^8^ cells/mL. The concentrated cells (300 μL), glass beads (0.3 g, 0.4–0.6 mm), PEG 6000 (30 μL, 50%, w: v) and 3 μg of *Not* I digested plasmid were mixed together in a 1.5-mL centrifuge tube and vortexed at the maximum speed for 15 s. The cell mixtures were transferred to 5-mL TAP and recovered for 18 h in the dark. The cells were then plated in a TAP plate supplemented with 10 μg/mL zeocin.

### Quantitative reverse transcription-PCR (qRT-PCR)

SYBR Premix Ex Taq Kits (TaKaRa) was used for qRT-PCR analysis. The ABI Prism 7900 Sequence Detection System was used for qRT-PCR to analyse cDNA (2 μL). The primers are listed in Additional file 4: Table S1, and the housekeeping gene *β*-*actin* was used as the reference. For each experiment, each sample was analysed in triplicates. The relative expression level of each gene was calculated using the 2^−ΔΔCt^ method [30].

The SYBR green RT-PCR method was used to quantify the levels of *C. reinhardtii c*-*crDOF* and *c*-*gDOF*, as well as 10 genes for lipid metabolism-related enzymes (*BCC1*, *ACP1*, *SQD1*, *SQD2*, *MGD1*, *DGD1*, *FAT*, *CIS1*, *ACS1*, and *PGP1*). The thermocycles were as follows: 40 cycles of 95 °C for 30 s, 95 °C for 5 s, and 60 °C for 31 s.

### Analysis of neutral lipids

Intracellular neutral lipids were stained with the fluorescent dye BODIPY 505/515. The droplets of neutral lipids in microalgae were observed with a laser scanning confocal microscope 710 (ZEISS, Germany). The specific experimental process is as follows: 200 μL of algae cells at the concentration of approximately 1.5 × 10^6^ cells/mL was collected and washed twice with 0.01 mol/L PBS buffer. After the addition of 2 μL of BODIPY505/515, the cells were incubated for 10 min in the dark, washed with 0.01 M PBS, and re-suspended in 200 μL of 0.01 mol/L PBS buffer. The BODIPY-stained algal cells were observed by a laser scanning confocal microscope with excitation at 488 nm and emission at 550 nm, at a gain of 550. The fluorescence intensity of BODIPY-stained algal cells was also recorded using a multifunctional microplate reader (SpectraMax L, Molecular Devices, Sunnyvale, CA, USA) to measure its intracellular neutral lipid content. Three replicates were set for each sample, and the measurements were repeated three times.

### Extraction and analysis of total cellular lipids

Algae were cultured and heat-induced as described above. After normal culturing for 48 h, the cells were collected and lyophilized. The lyophilized algal powder (5–15 mg) was placed in a glass centrifuge tube and mixed with an internal standard (50 μL of 500 μg/mL C19). Then, the sample was mixed with 1 mL of 2 M NaOH-CH_3_OH solution, placed in a shaker at 100 rpm for 1 h, and set for saponification at 75 °C for 15 min. After cooling down, 1 mL of 4 M HCl-CH_3_OH solution was added, and the pH was adjusted to below 2 with HCl, followed by incubation at 75 °C for 30 min. Subsequently, fatty acid methyl esters (FAMEs) were extracted with 1 mL of hexane three times. The upper phage was collected, filtered by a 0.22-m filter, dried with N_2_ and dissolved in 500 μL of methylene chloride. Three replicates were used for each sample.

The extracted fatty acid samples were analysed by GC–MASS (Thermo Polaris Q mass spectrometry equipped with a HP-5MS column, 30 mm × 0.25 mm, film thickness = 0.25 μm). The gas chromatography column was VF-23 ms (Part No. CP8827) with a maximum temperature of 260 °C and a size of 30.0 m × 320 μm × 0.25 μm. The GC inlet temperature was 250 °C, and high-purity He was the carrier gas. The mode was constant pressure, with the column head pressure at 1.2 psi and a split ratio of 10:1. The oven temperature for FAMEs analysis was as follows: 70 °C for 4 min, 25 °C/min to 195 °C, 3 °C/min to 205 °C, and 8 °C/min to 230 °C (hold for 1 min). The temperature in gas chromatograph-mass transfer line was 250 °C, and the mass spectrometer was set to full-scan mode. The peak was identified by matching the mass spectra of each compound with the National Institute of Standards and Technology mass spectral library. The data of fatty acids obtained were analysed with SPSS 13.0 software, and GraphPad Prism 5 was used for mapping.

## Additional files

**Additional file 1: Figure S1.** The comparison of the codon usage frequency of original Glycine max DOF (A) and optimised gDOF (B). The codon usage frequency is caculated by GCUA (http://gcua.schoedl.de/). The codon usage frequencies greater than 30, less than 29 and less than 9 are showed in black, red and grey, respectively.

**Additional file 2: Figure S2.** The intracellular neutral lipids content of DOF transformants after heat induction by bodipy staining method. The fluorescence intensity of BODIPY is positively correlated with the intracellular lipid content. Tran^c-crDOF^-12 and Tran^c-gDOF^-60 showed significantly higher lipids after heat induction for 24 h, 48 h and 72 h.

**Additional file 3: Figure S3.** Growth carves of transgenic algae Tran^c-crDOF^-12 and Tran^c-gDOF^-60 and control strain cc849. Transgenic algae presented similar growth whit control strian.

**Additional file 4: Table S1.** Complete list of primers used in this study.

**Additional file 5: Table S2.** The GenBank accession number of sequences used in Phylogenetic analysis.

## Abbreviations

DOF: DNA binding with one finger
qRT-PCR: quantitative reverse transcription-PCR
GC/MASS: gas chromatography/mass spectrometry
TAP: tris–acetate–phosphate
DCW: dry cell weight
BODIPY 505/515: 4,4-difluoro-1,3,5,7-tetramethyl-4-bora-3a,4a-diaza-s-indacen
FAMEs: fatty acid methyl esters
BCC1: acetyl-coA biotin carboxyl carrier 1
ACP1: acyl-carrier protein
FAT1: fatty acyl–acyl carrier protein thioesterase
ACS1: acetyl-CoA synthetase
CIS1: citrate synthase
SQD1: UDP-sulfoquinovose synthase
SQD2: sulfolipid synthase
MGD1: monogalactosyl diacylglycerol esters
DGD1: digalactosyl diacylglycerol synthase
PGP1: phosphatidyl glycerophosphate synthase
