# Supplementary material for: Understanding the functions of endogenous DOF transcript factor in Chlamydomonas reinhardtii
Source: Biotechnol Biofuels. 2019 Mar 27;12:67. doi: 10.1186/s13068-019-1403-1 (PMC6436238; doi:10.1186/s13068-019-1403-1)
Supplement: Supplementary file 1 — Additional file 1: Figure S1. The comparison of the codon usage frequency of original Glycine max DOF (A) and optimised gDOF (B). The codon usage frequency is caculated by GCUA (http://gcua.schoedl.de/). The codon usage frequencies greater than 30, less than 29 and less than 9 are showed in black, red and grey, respectively. [file 13068_2019_1403_MOESM1_ESM.doc]

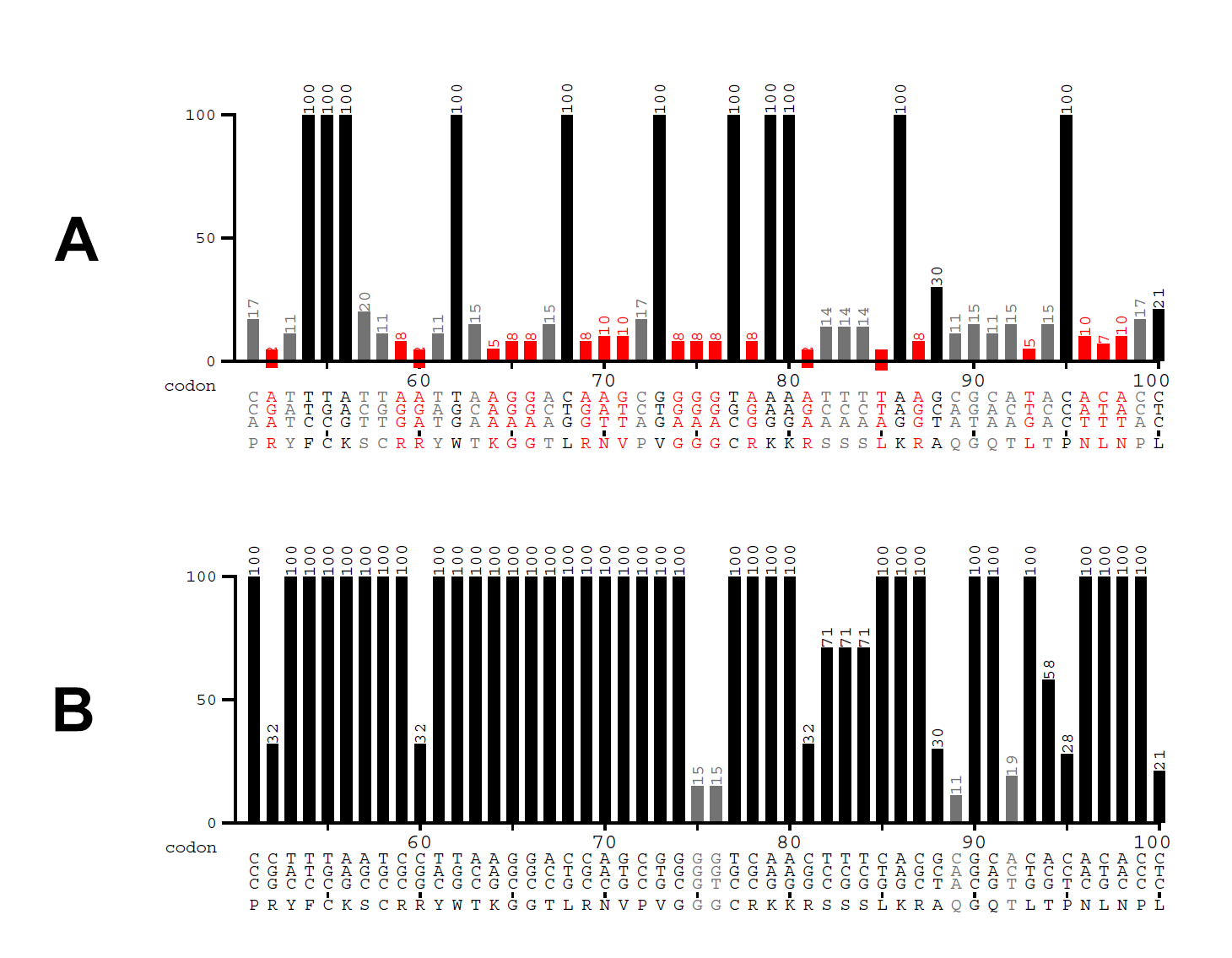


**Additional file 1: Figure S1. The comparison of the codon usage frequency of original Glycine max DOF (A) and optimized gDOF (B).** The codon usage frequency is caculated by GCUA(<http://gcua.schoedl.de/>). The codon usage frequencies greater than 30, less than 29 and less than 9 are showed in black, red and gray, respectively.
