# Supplementary material for: Understanding the functions of endogenous DOF transcript factor in Chlamydomonas reinhardtii
Source: Biotechnol Biofuels. 2019 Mar 27;12:67. doi: 10.1186/s13068-019-1403-1 (PMC6436238; doi:10.1186/s13068-019-1403-1)
Supplement: Supplementary file 2 — Additional file 2: Figure S2. The intracellular neutral lipids content of DOF transformants after heat induction by bodipy staining method. The fluorescence intensity of BODIPY is positively correlated with the intracellular lipid content. Tranc-crDOF-12 and Tranc-gDOF-60 showed significantly higher lipids after heat induction for 24 h, 48 h and 72 h. [file 13068_2019_1403_MOESM2_ESM.docx]

**Additional file 2: Figure S2. The intracellular neutral lipids content of DOF transformants after heat induction by bodipy staining method.** The fluorescence intensity of BODIPY is positively correlated with the intracellular lipid content. Tran^c-crDOF^-12 and Tran^c-gDOF^-60 showed significantly higher lipids after heat induction for 24h, 48h and 72h.
