# Supplementary material for: Understanding the functions of endogenous DOF transcript factor in Chlamydomonas reinhardtii
Source: Biotechnol Biofuels. 2019 Mar 27;12:67. doi: 10.1186/s13068-019-1403-1 (PMC6436238; doi:10.1186/s13068-019-1403-1)
Supplement: Supplementary file 3 — Additional file 3: Figure S3. Growth carves of transgenic algae Tranc-crDOF-12 and Tranc-gDOF-60 and control strain cc849. Transgenic algae presented similar growth whit control strian. [file 13068_2019_1403_MOESM3_ESM.docx]

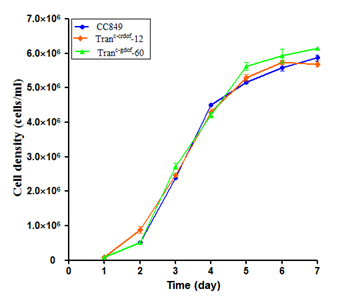


**Additional file 3: Figure S3 Growth carves of transgenic algae Tran^c-crDOF^-12 and Tran^c-gDOF^-60 and control strain cc849**. Transgenic algae presented similar growth whit control strian.
