# Supplementary material for: Understanding the functions of endogenous DOF transcript factor in Chlamydomonas reinhardtii
Source: Biotechnol Biofuels. 2019 Mar 27;12:67. doi: 10.1186/s13068-019-1403-1 (PMC6436238; doi:10.1186/s13068-019-1403-1)
Supplement: Supplementary file 4 — Additional file 4: Table S1. Complete list of primers used in this study. [file 13068_2019_1403_MOESM4_ESM.doc]

**Additional file 4: Table S1.** Complete list of primers used in this study.

| **Primer name** | **PRIMER SEQUENCE(5’-3’)** | **description** |
| --- | --- | --- |
| c-crdof-F | cacgtgCATGGTAGACGGTGGTTCG | Primers used for amplification of *crDOF* |
| c-crdof-R | ggtaccTTCACCTAGCACCCGAGTAA |
| c-gdof-F | cacgtgATGGGCAAGCCGATCCCCA | Primers used for amplification of *gDOF* |
| c-gdof-R | ggtaccTTACATCAGCGGGGACTGG |
| ActinF | ACCCCGTGCTGACTG | qRT-PCR primer of reference gene β-actin |
| ActinR | ACGTTGAAGGTCTCGAACG |
| QACSF | CGTCGGATACTGGCAGGGTG | qRT-PCR primer of *ACS1*expression in transgenetic algae |
| QACSR | ATAAGCCGGGACAGGAAGC |
| QcrdofF | CACATGCATGGTAGACGGTGGTTCG | qRT-PCR primer of *crDOF* expression in transgenetic algae |
| QcrdofR | GGTACCTTCACCTAGCACCCGAGTAA |
| QgdofF | GCCAGACTCTGACGCCTAAC | qRT-PCR primer of *gDOF* expression in transgenetic algae |
| QgdofR | CCAGGATGTCGCAGAACG |
| QbccF | TTGAGGCTGCTACCAACGG | qRT-PCR primer of *BCC1* expression in transgenetic algae |
| QbccR | TCTTCAGCTCGAACTCGACAAT |
| QacpF | TTCGCTGTCCTGATTTCTTC | qRT-PCR primer of *ACP1* expression in transgenetic algae |
| QacpR | ACGGCTGATTATGTTGGTGA |
| QfatF | AGCGATGGACGGTTGTGG | qRT-PCR primer of *FAT11* expression in transgenetic algae |
| QfatR | TCGGGTCTGGAGGTTGC |
| QcisF | ATGAGAACCCGGAACCCTT | qRT-PCR primer of *CIS* expression in transgenetic algae |
| QcisR | CGCAGCGATTGATTGACG |
| Qsqd1F | AGGGCTACATCACCATCAACCAC | qRT-PCR primer of *SQD1* expression in transgenetic algae |
| Qsqd1R | TGGTAGAAAGAGTTGCCCTGCTTG |
| Qsqd2F | GCATGGGTTCGCTGTGG | qRT-PCR primer of *SQD2* expression in transgenetic algae |
| Qsqd2R | TCTGCGGTGTCGTCGTGT |
| QmgdF | CGTCGGATACTGGCAGGGTG | qRT-PCR primer of *MGD1* expression in transgenetic algae |
| QmgdR | AAGATAAGCCGGGACAGGAAGC |
| QdgdF | TCGCCGACGCATTGG | qRT-PCR primer of *DGD1* expression in transgenetic algae |
| QdgdR | CGCCGTCTGCTTTGCTC |
| QpgpF | GTTATCTTCATCGCCGCCTCAG | qRT-PCR primer of *PGP1* expression in transgenetic algae |
| QpgpR | ACAGTCGCGATCTTGAGCTTTC |

underlined bases indicate the restriction enzyme sites
