# Supplementary material for: Understanding the functions of endogenous DOF transcript factor in Chlamydomonas reinhardtii
Source: Biotechnol Biofuels. 2019 Mar 27;12:67. doi: 10.1186/s13068-019-1403-1 (PMC6436238; doi:10.1186/s13068-019-1403-1)
Supplement: Supplementary file 5 — Additional file 5: Table S2. The GenBank accession number of sequences used in Phylogenetic analysis. [file 13068_2019_1403_MOESM5_ESM.doc]

Additional file 5: Table S2 The GenBank accession number of sequences used in Phylogenetic analysis

| **Species** | **Accession NO.** |
| --- | --- |
| ***Chlamydomonas reinhardtii*** | XP_001696918.1 |
| ***Glycine max*** | DQ857261.1 |
| ***Glycine soja*** | KHN01870.1 |
| ***Medicago truncatula*** | XP_013459285.1 |
| ***Sorghum bicolor*** | AGL40009.1 |
| ***Jatropha curcas*** | NP_001295662.1 |
| ***Volvox carteri*** | EFJ40859.1 |
| ***Picea abies*** | AAY28423.1 |
| ***Ipomoea batatas*** | BAH58100.1 |
| ***Prunus dulcis*** | ALI97581.1 |
| ***Platycodon grandiflorus*** | AEF13372.1 |
| ***Hordeum vulgare*** | CAJ29308.1 |
| ***Nicotiana tabacum*** | BAQ19266.1 |
| ***Theobroma cacao*** | EOY03487.1 |
| ***Populus trichocarpa*** | EEE91676.1 |
| ***Ostreococcus tauri*** | XM_003078771.1 |
